# Janus kinase inhibitors – a role for the treatment of cutaneous T-cell lymphomas?

**DOI:** 10.3389/or.2025.1482866

**Published:** 2025-08-11

**Authors:** Sarah E. Packer, Patrick M. Brunner

**Affiliations:** Department of Dermatology, Icahn School of Medicine at Mount Sinai, New York, NY, United States

**Keywords:** CTCL, lymphoma, mycosis fugoides, Sezary syndrome, JAK inhibition

## Abstract

Despite increases in prevalence, many cutaneous T-cell lymphoma (CTCL) patients still lack effective and safe therapies for their disease. The most prevalent subtype, mycosis fungoides is usually managed with skin directed treatments in early stages, while advanced stages are often targeted with systemic medications. These treatments are all symptomatic except for allogeneic hematopoietic stem cell transplantation, which is associated with its own risks of relapse and potentially fatal complications. A novel class of drugs termed “JAK inhibitors” (JAKi) has recently been developed primarily for chronic inflammatory diseases, but there is substantial evidence of JAK/STAT pathway overactivation also in CTCL. As of 1 December 2024, 14 JAKis have been collectively approved by the European Medicines Agency, the Food and Drug Administration and the Pharmaceutical and Medical Devices Agency of Japan. Despite some evidence from case reports, the efficacy and safety of JAKi in CTCL remains to be determined in controlled clinical trials. This review summarizes the current evidence on pathogenic JAK activation and its potential therapeutic inhibition in CTCL.

## Introduction

Cutaneous T-cell lymphomas (CTCL) are a diverse group of non-Hodgkin lymphomas originating from malignant skin T cells. Although exact estimates fluctuate, a growing body of literature supports an increase in CTCL cases over the last years (1–4). CTCL is more commonly diagnosed in males, individuals of black race, and older adults (1, 2, 4–12, 13). From 2002 to 2021, CTCL incidence was 1.7 times higher in black individuals and males (compared to whites and females respectively), and 4.5 times higher in those aged 65–74 compared to 20–54 years (14). There are many CTCL subtypes, which differ in prevalence, clinical manifestation, and severity (1, 8). Mycosis fungoides (MF) and Sézary syndrome (SS) are the most prominent subtypes and typically stem from malignant mature CD4^+^ helper T cells. Representing approximately half of all CTCL cases, MF is the most prevalent subtype and generally presents with a more indolent course than SS. SEER18 data (covering ~28% of the U.S. population) reported that MF and SS respectively accounted for 56.6% and 1.8% of CTCL cases (n=14,942) from 2000 to 2018 (1). More recent SEER22 data (~48% of the U.S. population) estimate that MF and SS comprise 37.8% and 1.4% of CTCL cases (n=46,433), respectively (14). To assess disease severity and prognosis, MF and SS are staged (IA-IVB) based on ISCL/EORTC guidelines using the TNMB system, which considers skin lesions, and involvement of the lymph nodes, viscera, and blood (8, 16). Skin involvement includes T1 (<10% patches/plaques), T2 (>10% patches/plaques), T3 (tumors), and T4 (erythroderma) (16, 17). Lymph node involvement spans from N0 to N3, and metastasis involves M0 (none) and M1 (visceral involvement). Blood involvement spans from B0 to B2. Overall, early-stage disease encompasses stages IA, IB and IIA, while advanced stage disease includes stages IIB, III, IVA and IVB (7, 16). Approximately 70% of MF cases present with early-stage disease (18–20). Five-year overall survival (OS) rates are high for early‐stage disease with rates of 96-100% for stage IA, 73‐86% for stage IB, and 49‐73% for stage IIA (21). However, OS rates drop significantly in advanced stages, including IIB (40‐65%), III (40-57%), IVA (15‐40%) and IVB (0‐15%) (21). Therapeutically, early-stage CTCL is often managed with topical steroids, phototherapy, and sometimes irradiation (134, 135). On the other hand, advanced-stage disease typically requires systemic treatments (134, 23).

## Established treatment approaches

Depending on disease severity, established treatments for CTCL range from skin-directed therapies for early-stage disease to systemic therapies for advanced-stage disease (134, 17). These therapies are generally symptomatic rather than curative. Allogeneic hematopoietic stem cell transplantation (allo-HSCT) remains the only curative option, but it is associated with high relapse rates and serious complications, including graft-versus-host disease (GVHD), limiting its use to carefully selected patients (24, 25).

### Topicals

Topical corticosteroids (TCS) are a common first-line treatment for early-stage MF, achieving meaningful clinical responses, although long-term efficacy data are limited (26, 27). In a 1998 prospective study, Zackheim et al. reported response rates of 94% (T1) and 82% (T2) in 79 patients with patch (n=75) or plaque stage (n=4) MF. Complete remission occurred in 63% of T1 and 25% of T2 cases after 3–4 months (28). In a 2021 retrospective analysis, Kartan et al. observed disease improvement in 73% of 37 MF patients, with complete remission in 44% of responders after 18.5 months on average (27). However, TCS are typically inadequate for higher stage disease (IIA and above), with only 33% of patients responding (27). A primary limitation of TCS is the risk of cutaneous atrophy with extended use (29). Topical chlormethine/mechlorethamine, a chemotherapy agent, offers an alternative with 76.7% of MF patients (n=206; stage IA and IB) achieving partial response in a real-world setting (30).

### Phototherapy

Phototherapy, most commonly psoralen plus UVA (PUVA) or narrowband UVB (NB-UVB), is a standard treatment for early-stage MF (31). Complete remission rates range from 60–81% for NB-UVB and 62–71% for PUVA. However, PUVA increases the risk of skin cancers, including melanoma, and is less widely available (31, 32).

### Radiotherapy

High-energy radiation therapy is used to target malignant cells in individual lesions or the entire body as in total skin electron-beam therapy (TSEB) (33). Radiation therapy, particularly TSEB, can achieve average complete response rates of 81%. However, relapse rates are high, with up to 73% of patients relapsing within five years post-treatment (34–46). Repeated courses of high-dose TSEB increase the risk of cumulative adverse effects. (34–36, 38–41, 47–49). Low-dose radiation therapy (RT) (7-12 Gy) has been utilized as an alternative because it is associated with fewer grade 2 (33% vs. 79%) and grade 3 adverse events (6% vs. 15%) compared to a standard dose (30 Gy) (50). However, low-dose RT (10 to <20 Gy) demonstrates diminished efficacy, achieving complete responses in only 35% of patients compared to 62% in standard dosing (>30 Gy) (51).

### Small molecule inhibitors

Small molecule inhibitors (SMIs) frequently used for CTCL treatment include methotrexate, bexarotene, and HDAC inhibitors. Methotrexate (MTX), a folic acid metabolism inhibitor effective in highly proliferative cells, has been used to treat CTCL (52, 53). Oral MTX achieved complete responses in 30% of MF and 5.5% of SS cases, though 57% of responders relapsed after a median time of 11 months after treatment implementation (53, 54). Bexarotene, a synthetic retinoid, is used in patients with refractory advanced-stage MF (52). When administered orally (initial dose: 300 mg/m^2^/day), bexarotene prompted response rates of 54% in early-stage MF patients (n=28) and 45% in advanced-stage MF patients (n=56) (55, 56). Bexarotene doses above 300 mg/m^2^/day produced response rates of 67% and 55% in early and advanced MF respectively (55, 56). Two histone deacetylase (HDAC) inhibitors are currently approved to treat CTCL by the FDA. Vorinostat, a hydroxamic acid, inhibits class I and II HDACs in patients with refractory or relapsed CTCL (57, 58). Romidepsin is a bicyclic, class I HDAC inhibitor approved to treat relapsed CTCL (59, 60).

### Monoclonal antibodies

Approved monoclonal antibody (mAb) therapies for CTCL include mogamulizumab and brentuximab vedotin. Mogamulizumab is a defucosylated, humanized IgG1 anti-CCR4 mAb that promotes antibody-dependent cellular cytotoxicity (ADCC) to deplete target cells in patients with relapsed or refractory MF or SS (61, 62). Brentuximab vedotin is an anti-CD30 antibody-drug conjugate used to treat CD30+ PTCL and CTCL, including refractory, CD30+ transformed MF (63, 64). Both antibodies demonstrated superior efficacy compared to conventional therapies in controlled trials (65, 66), making them important additions to the therapeutic armamentarium for CTCL.

### Allogeneic hematopoietic stem cell transplantation

Allogeneic hematopoietic stem cell transplantation (allo-HSCT), the sole curative measure for MF/SS, is generally used for patients with progressive stage IIB-IV CTCL (including transformed MF) (67, 68). A meta-analysis by Iqbal et al. reported pooled progression-free survival (PFS) and relapse rates of 36% and 47%, respectively (24). Graft-versus-host disease (GVHD) occurs in 40%–60% of patients undergoing allo-HSCT and represents a common, significant adverse event (25) associated with considerable morbidity and mortality (69, 70).

Despite all these treatment options for CTCL, many patients encounter disease relapses, mandating more efficacious, highlighting the need for safer, more effective treatment options for long-term disease control

## JAK inhibitors – a novel group of therapeutics for inflammatory and malignant skin diseases

The JAK/STAT signaling pathway plays a key role in regulating immune responses and inflammation, and its dysregulation has been implicated in the pathogenesis of CTCL (71–74). This evolutionarily conserved pathway promotes gene expression following cytokine receptor engagement by interleukins (ILs), interferons (IFNs), and growth factors (75). JAK/STAT signaling is essential for orchestrating both innate and adaptive immune responses to pathogens and malignancies (76–78). All four known JAK family members (JAK1, JAK2, JAK3 and TYK2) share conserved domains (kinase domain, pseudokinase domain, SH2 domain and FERM domain) (74). However, they respond to different cytokines based on their unique tyrosine phosphorylation sites (74). The STAT family includes seven molecules (STAT1, STAT2, STAT3, STAT4, STAT5a, STAT5b, and STAT6) (74). JAKs recruit and phosphorylate one or more STATs that then dimerize and translocate into the nucleus (74). STATs act as transcription factors (TFs) to promote expression of genes involved in angiogenesis, proliferation, cell differentiation and apoptosis (79). Dysregulation of JAK/STAT signaling has been implicated in autoimmune diseases and cancers, including hematologic malignancies such as CTCL (74, 80, 81). In addition to its role in tumorigenesis and metastasis of various cancers (82 83, 84, 86, 87), JAK/STAT overactivation has been specifically implicated in hematologic malignancies, including CTCL (88). However, the precise role of JAK inhibitors (JAKis) in CTCL remains to be clarified.

**FIGURE 1:**
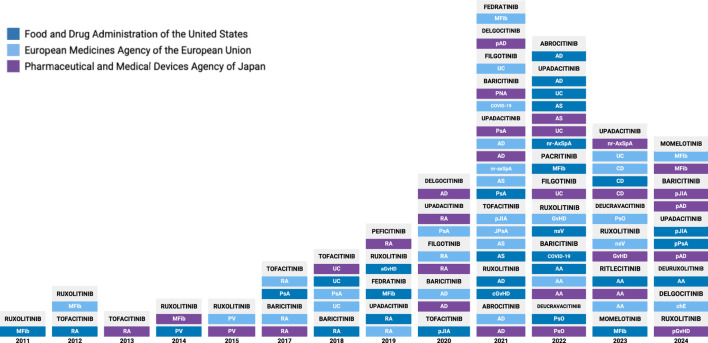
Timeline of JAK Inhibitor approvals by global regulatory agencies. AA, Alopecia areata; AD, Atopic dermatitis; aGvHD, acute graft-vs-host disease; AS, Ankylosing spondylitis; cGvHD, chronic graft-vs-host disease; COVID, Coronavirus-2019; CD, Crohn’s Disease; chE, chronic hand eczema; GvHD, graft-vs-host disease; JIA, juvenile idiopathic arthritis; JPsA, juvenile psoriatic arthritis; MFib, myelofibrosis; pJIA, polyarticular juvenile idiopathic arthritis; nr-AxSpA, non-radiographic axial spondyloarthritis; nsV, nonsegmental vitiligo; pAD, pediatric atopic dermatitis; pGvHD, pediatric graft-vs-host disease; PsA, psoriatic arthritis; PsO, psoriasis; pPsA, pediatric psoriatic arthritis; PNA, Pneumonia (COVID-19 induced); PV, polycythemia vera; RA, rheumatoid arthritis; UC, ulcerative colitis.

In 2011, the Food and Drug Administration (FDA) approved its first JAKi, ruxolitinib, to treat myelofibrosis (89). Since then, new JAK inhibitors continue to be approved each year by the FDA and other regulatory agencies. As of 1 December 2024, 14 JAKis have been approved by respective administrative bodies in the United States, the European Union and Japan across 17 different indications (Figure 1) (90–92). These include chronic inflammatory skin conditions (atopic dermatitis, psoriasis, chronic hand eczema, nonsegmental vitiligo), arthritis and spondyloarthritis (rheumatoid arthritis, psoriatic arthritis, ankylosing spondylitis, non-radiographic axial spondyloarthritis), autoimmune and pediatric conditions (alopecia areata, graft versus host disease, polyarticular juvenile idiopathic arthritis, juvenile psoriatic arthritis), myeloproliferative neoplasms (myelofibrosis, polycythemia vera), inflammatory bowel diseases (ulcerative colitis, Crohn’s disease), and infectious disease (COVID-19 induced pneumonia) (Table 1).

**TABLE 1:** Approved JAK inhibitors by indication and regulatory body from 2011 to 2024.

Approved Drug	Indication (Government Agency)
Abrocitinib	Atopic Dermatitis (EMA, FDA, and PMDA)
Baricitinib	Alopecia Areata (EMA, FDA, and PMDA)
	Atopic Dermatitis (EMA and PMDA)
	COVID (FDA and EMA) / COVID-19 Induced Pneumonia (PMDA)
	Polyarticular Juvenile Idiopathic Arthritis (PMDA)
	Rheumatoid Arthritis (EMA, FDA, and PMDA)
Delgocitinib	Atopic Dermatitis (PMDA)
	Chronic Hand Eczema (EMA)
Deucravacitinib	Psoriasis (EMA, FDA, and PMDA)
Deuruxolitinib	Alopecia Areata (FDA)
Fedratinib	Myelofibrosis (EMA and FDA)
Filgotinib	Rheumatoid Arthritis (EMA and PMDA)
	Ulcerative Colitis (EMA and PMDA)
Momelotinib	Myelofibrosis (EMA, FDA, and PMDA)
Pacritinib	Myelofibrosis (FDA)
Peficitinib	Rheumatoid Arthritis (PMDA)
Ritlecitinib	Alopecia Areata (EMA, FDA, and PMDA)
Ruxolitinib	Acute Graft versus Host Disease (FDA)
	Atopic Dermatitis (FDA)
	Chronic Graft versus Host Disease (FDA)
	Graft versus Host Disease (EMA and PMDA)
	Myelofibrosis (EMA, FDA, and PMDA)
	Nonsegmental Vitiligo (FDA and EMA)
	Polycythemia Vera (EMA, FDA, and PMDA)
Tofacitinib	Ankylosing Spondylitis (EMA and FDA)
	Juvenile Psoriatic Arthritis (EMA)
	Polyarticular Juvenile Idiopathic Arthritis (EMA and FDA)
	Psoriatic Arthritis (EMA, FDA)
	Rheumatoid Arthritis (EMA, FDA, and PMDA)
	Ulcerative Colitis (EMA, FDA, and PMDA)
Upadacitinib	Ankylosing Spondylitis (EMA, FDA, and PMDA)
	Atopic Dermatitis (EMA, FDA, and PMDA)
	Crohn's Disease (EMA, FDA, and PMDA)
	Non-Radiographic Axial Spondylarthritis (EMA, FDA, and PMDA)
	Psoriatic Arthritis (EMA, FDA and PMDA)
	Polyarticular Juvenile Idiopathic Arthritis (FDA)
	Rheumatoid Arthritis (EMA, FDA, and PMDA)
	Ulcerative Colitis (EMA, FDA, and PMDA)

EMA, European Medicines Agency; FDA, Food and Drug Administration; PMDA, Pharmaceutical and Medical Devices Agency of Japan.

## JAK inhibition in CTCL–a viable concept?

In recent years, sequencing techniques (including whole exome sequencing, whole genome sequencing, and targeted capture sequencing) have been utilized to investigate JAK-STAT genomic alterations underlying MF and SS (79, 93–104). A review by Garcia-Diaz et al. evaluating NGS publications (88, 96–98, 100, 101, 103–106) in 2021 found that ≥60% of cases showed genetic alterations in JAK/STAT genes (79). STAT3 and STAT5B amplifications were fairly common (60% of patients), in contrast to activating JAK mutations (4%) (79). In addition to missense mutations reported by Song et al. (5% of 55 MF; 3% of 31 SS), amplifications in STAT5B were noted, for instance, by Choi et al. (62.5% of 40 CTCL) and Iyer et al. (18% of 49 MF samples) (93, 98, 100, 104). Missense STAT3 mutations were reported by Song et al. (15% of 55 MF) (93). Amplifications, gain of function mutation or SNVs of STAT3 were observed by Iyer et al. (12% of 49 MF samples), Kiel et al. (3% of 66 SS), and Bastidas-Torres et al. (11% of 9 MF), amongst others (97, 98, 100, 101). STAT5A alterations were noted, for example, by McGirt et al. (103) and Vaqué et al. (96), in addition to STAT1 (96). Wang et al. also noted significant upregulation of STAT1 in SS patients (94). Ligand-independent (constitutive) phosphorylation and activation has respectively been observed in STAT5 (95) and STAT3 (102).

Regarding the JAK family, reports of JAK1 amplifications or gain of function mutations included Iyer et al. (27% of 49 MF) and Kiel et al. (3% of 66 SS) (93, 97, 98, 100). Song et al. identified missense JAK1 mutations in 9% of 55 MF, and Vaqué et al. described a tolerated missense mutation in 1/11 MF cases (93, 96). JAK3 alterations included amplifications and deleterious SNVs in 22% of 9 MF tumors by Bastidas-Torres et al. (101), as well as various mutations observed by Iyer et al. (12% of 49 MF) (100), Koo et al., (35.4% of 65 CTCL) (95), and Kiel et al. (3% of 66 SS) (97). Additional JAK3 tumor SNVs were noted by Woollard et al., as well as missense and multi-hit mutations by Song et al. (13% MF) (93, 98). JAK2 showed focal amplification in 12.5% of 40 CTCL by Choi et al. (104) and 4% of 49 MF by Iyer et al. (100). Woollard et al. reported mixed JAK2 alterations including deletions, amplification, and SNVs (98).

Multiple members of the SOCS (Suppressor of Cytokine Signaling) family, key negative regulators of the JAK/STAT pathway, were recurrently altered, suggesting a loss of pathway inhibition may contribute to malignant T-cell survival. Specifically, SOCS1 deletions were noted in 33% of 27 MF (extension cohort) by Bastidas-Torres et al. (101). SOCS2 deletions were identified in CTCL cases by Choi et al. (104). Vaqué et al. noted a deleterious missense mutation in SOCS5 in 1/11 MF samples (96). SOCS7 showed alterations in SS (Woollard et al.) (98), with additional deletions and mutations reported by Song et al. (93). Mutations in those negative JAK-STAT regulators highlight frequent impairment of negative feedback mechanisms in this pathway. Such an increase in activity of the JAK/STAT pathway might directly promote tumor growth, but will likely also depend on the cellular context within the tumor microenvironment (TME) (109). While the mechanisms underlying CTCL and its progression are still only incompletely understood, differences within the TME are believed to distinguish early and advanced MF. Driven by Th1 (CD4^+^ helper T cells), Tc1 (CD8^+^ cytotoxic T cells) and NK (natural killer) cells, the early MF TME promotes type 1 immune skewing (IL-12 and IFN-y) and anti-tumor cytotoxic activity via STAT1 and STAT4 (110, 111). In contrast, the tumorigenic TME of advanced-stage MF utilizes STAT3, STAT5 and STAT6 signaling pathways to promote type 2 inflammation via cytokines (IL-4, IL-5, IL-13) and chemokines (CCL17, CCL18, CCL22, CCL26) (110, 112), but exact mechanisms remain unclear. Thus, clinical trials of JAK inhibitors are necessary to distinguish true disease drivers from bystander or counterregulatory JAK activation in CTCL.

### Published case reports

In addition to sequencing results supporting JAK/STAT amplification in CTCL, JAKis have been administered to several CTCL patients as detailed in a few published case reports and clinical trials. Case reports by Castillo et al., Kook et al. and Mo et al. detail noticeable improvement in MF following treatment with systemic upadacitinib (114–116). Similarly, Levy et al. noted improvements to symptoms following initiation of another JAKi, ruxolitinib (117).

Castillo et al. reported a significant clinical response to 15 mg upadacitinib QD in an 87-year-old male with erythrodermic MF (stage 3; T4N0M0B0) and severe pruritus (114). Following at least 6 weeks of treatment (sequential manner: cyclosporine, methotrexate, dupilumab, acitretin and narrowband UV-B therapy), the patient demonstrated no significant response and was started on 15 mg upadacitinib QD for 16 weeks. After 16 weeks of upadacitinib, the patient demonstrated noticeable improvement in generalized itching and redness, as well as diminished scaling (from >80% body surface area to <10% post treatment). Improvements to the abdomen and pubic area (resolved erythematous scaly patches and plaques), as well as the back (diminished erythema and scaling) were noted.

Kook et al. reported significant clinical improvement in a 43-year-old male diagnosed with MF (Stage IB; onset 7 years prior) predominantly on the trunk and lower back (115). The patient was initially misdiagnosed with AD. Following proper diagnosis of MF, NB-UVB therapy and methotrexate (20 mg) were initiated. After 2 months of treatment, the patient had not improved and was started on 15 mg upadacitinib QD for 16 weeks. After 1 week, the patient noted noticeable improvement in perceived pain in addition to improved itch demonstrated by a decrease in Numerical Rating Scale (range 0–10) score from 9 to 3.

Levy et al. treated a 16-year-old male patient with recurrent subcutaneous panniculitis-like T-cell lymphoma (SPTCL) and hemophagocytic lymph histiocytosis (HLH) (117). From age 11 to 16, the patient repeatedly relapsed during various therapies (including corticosteroids, cyclosporine A, etoposide, anakinra and methotrexate). The patient was started on 15 mg ruxolitinib BID and achieved remission after 4 months. Ruxolitinib was ultimately discontinued for 8 months until disease recurred. 10 months following re-initiation of ruxolitinib monotherapy, the patient remained in complete remission.

Mo et al. report a 44-year-old male previously diagnosed with severe generalized eczema and treated with TCS and phototherapy (116). With no symptom improvement noted, he was started on upadacitinib and exhibited partial relief of symptoms. After approximately 7 months, upadacitinib was stopped and rapid deterioration of symptoms was observed, including debilitating generalized pruritus and redness. The patient was ultimately diagnosed with folliculotropic mycosis fungoides and unsuccessfully treated with oral methotrexate, TCS and oral prednisone. With no initial biopsy performed, it is uncertain whether the mycosis fungoides was present and treated successfully with upadacitinib. It is notable, however, that introduction of this JAKi provided symptom relief for this patient until it was ultimately discontinued.

### Clinical trials

A clinical trial initiated by Wilcox et al. (NCT03601819) evaluated the JAK2 inhibitor pacritinib in CTCL (118). In this open label phase Ib study, the primary endpoint was the dose limiting toxicity (DLT) rate during the 1st cycle (28 days) of pacritinib (118). However, given low accrual of participants as per ClinicalTrials.gov, this trial was terminated early and does not have published results (118).

Horwitz et al. completed a non-randomized, open-label phase 1/2a clinical trial (multi-dose and multi-center) (NCT01994382) investigating the efficacy of cerdulatinib in patients with relapsed or refractory PTCL (n = 65) and CTCL (n = 41) (119). Initiated in August 2013, the study was completed in December 2020 with complete results provided April 2022 (120). Cerdulatinib, an oral small-molecule inhibitor of spleen tyrosine kinase (SYK), JAK1 and JAK3, caused adverse events (AEs) in 100% of PTCL and CTCL patients (120). Serious AEs were noted in 65% of PTCL patients and 51% of CTCL patients (136). In PTCL, common (n ≥ 3) serious AEs were diarrhea, pyrexia, pneumonia, sepsis and neoplasm progression (120). In CTCL, common (n ≥ 2) serious AEs were diarrhea, pneumonia, sepsis, staphylococcal bacteremia, dehydration, and neoplasm progression (120). In phase 2, overall response rates (ORR; both complete and partial responses) were observed in 36% of PTCL patients evaluated (n = 58). (120) Of the PTCL subtypes, patients with angioimmunoblastic T-cell lymphoma/T follicular helper lymphoma (AITL/TFH; n = 29) demonstrated the highest ORR (52%) and a clinical benefit (CB) of 63% (120). The other-PTCL (n = 25) cohort had an ORR of 32% and 73% CB. Lastly, PTCL-NOS (not otherwise specified; n = 11) exhibited 0% ORR and 22% CB. Out of 41 CTCL patients who were started on cerdulatinib, 37 were evaluated for efficacy in 2019 (121). Notably, MF patients exhibited higher ORR (45%) and CR (9%) compared to SS patients (17% ORR; 0% CR) (121).

In November 2016, Moskowitz et al. initiated a non-randomized, open-label Phase 2 clinical trial (multi-center) (NCT02974647) investigating the efficacy of 20 mg BID ruxolitinib in patients with relapsed/refractory peripheral T-cell lymphomas (PTCLs; n = 45) or MF (n = 7). While participant recruitment is ongoing as of 16 January 2025, trial completion is estimated for November of 2025 (126). Preliminary data on the initial cohort (n = 52) was published in Blood in 2021 (122). Common adverse events included febrile neutropenia, fatigue, diarrhea, anemia, as well as decreases in platelet and neutrophil counts (122). All patients were assigned to one of three cohorts based on the presence of JAK/STAT mutations (n = 21), pSTAT3 expression (≥30%; n = 14) or the absence of both (n = 17; 6 presented with incomplete sequencing data). The most common mutated genes during next-generation sequencing were STAT5B (n = 8), STAT3 (n = 7) and JAK3 (n = 6). PTCL patients (n = 19) with JAK/STAT mutations (cohort 1) exhibited clinical benefit rates (CBRs) approximately four times higher (53%) than those negative for biomarker expression (13% for cohort 3; n = 15) (122). Notably, CBRs of patients with JAK/STAT alterations (cohort 1 & 2) were significantly higher (p = 0.02) than those without (cohort 3) (122). While not all biomarker-positive PTCL patients responded to treatment, this result supports the role of JAK/STAT alterations in T-cell lymphomas, including CTCL. Conclusions about ruxolitinib efficacy in MF are limited in this study by the small cohort utilized. Although 71% of all MF cases presented with JAK/STAT mutations or pSTAT3 expression, clinical efficacy was limited (CBR = 14%) (122). These data suggest a potential for subtype specific JAK/STAT involvement and thus consideration for future treatment selection.

Moskowitz et al. are currently conducting a Phase I open-label multi-center clinical trial (NCT05010005) evaluating ruxolitinib in combination with duvelisib (phosphoinositide 3-kinase inhibitor) in patients with relapsed or refractory T- or NK-cell (natural killer cell) lymphoma (n = 49) (124). Patients are to receive 20 mg ruxolitinib BID with duvelisib (25 mg, 50 mg or 75 mg BID). The study includes dose escalation to determine maximum tolerated dose and efficacy evaluation in two cohorts (with and without JAK/STAT pathway activation). The study initiated on 12 August 2021, has terminated recruitment and estimates completion to occur in August 2027 (124). Preliminary results (2024) noted a maximum tolerated dose of 20 mg of ruxolitinib and 25 mg of duvelisib BID (127). The overall response rate (ORR) and complete response rate (CR) of all enrolled patients (n = 49) was 41% and 24% respectively (127). In the JAK/STAT activation cohort, complete responses (29%) occurred twice as often and overall response ratios (52%) were four times greater than those without mutations (14%; 14%) (p = 0.023) (127). Enrolled patients (n=49) presented with various disease subtypes, including T-follicular helper lymphomas (TFH; n = 14), PTCL-NOS (n = 13), and MF (n = 7) (127). The highest ORR (79%) and CR (64%) were observed in patients with TFH lymphomas (127). PTCL-NOS patients exhibited an ORR and CR of 23% and 15% respectively (127). Notably, MF patients had the lowest benefit, with an ORR of 14% and no complete responses (127). Treatment related AEs (grade 3 through 5) included neutropenia (24% G3, 14% G4), anemia (16% G3), thrombocytopenia (6% G3, 6% G4), lung infections (4% G3), hypertension (4% G3), hypertriglyceridemia (4% G3), transaminitis (4% G3), sepsis (2% G3, 2% G5), urinary tract infection (2% G3), diarrhea (2% G3), weight gain (2% G3), leukopenia (2% G3), and mucositis (2% G3) (127).

Wilcox et al. are currently conducting a non-randomized Phase 2, open label, clinical trial (NCT04858256) evaluating pacritinib in patients with relapsed/refractory T-cell neoplasms (goal n = 100) (125). All patients will receive 200 mg BID pacritinib and be grouped by subtype (PTCL-NOS, AITL/TFH PTCL, CTCL, Other PTCL). ORR, the primary outcome measure, will be assessed in CTCL by using the modified Severity Weighted Assessment Tool (mSWAT).

Brunner et al. are currently conducting a Phase 2A, open-label, clinical trial (NCT05879458) evaluating ritlecitinib in patients with CTCL including MF and SS (estimated n = 20) (123). All patients will receive 200 mg QD for 8 weeks followed by 100 mg QD for 16 weeks. The primary endpoint is the change in mSWAT at week 24 compared to baseline. Secondary endpoints include safety and quality of life measures.

## JAKi use in CTCL – benefits, limitations and practical applications

Given the abovementioned reasons, JAKis might offer a targeted therapeutic strategy for CTCL. JAKis have exhibited efficacy in improving pruritus, erythema and tumor burden for some patients with advanced or refractory disease. This suggests a potential added benefit of JAKi use in patients who have previously failed traditional therapies. Given that existing evidence is based on a handful of case reports and few clinical trials, it remains speculative whether JAK inhibition will have a role in CTCL treatment, especially when considering specific disease subsets. More data is necessary to further clarify JAKi efficacy in CTCL. JAKi side effects are relatively well characterized in chronic inflammatory diseases and can include opportunistic infections and herpes zoster reactivation, hematologic toxicities (anemia, thrombocytopenia and neutropenia), acne, thromboembolic events, gastrointestinal problems (diarrhea and nausea), liver enzyme elevations, dyslipidemia, fatigue and headache (128). The safety profile in CTCL, however, remains to be determined with robust data from more clinical trials.

## Future research directions

To determine a role for JAKi in CTCL treatment, more controlled clinical trials are evidently necessary, that include clinical endpoints such as mSWAT, quality of life and biomarkers from the blood and skin that might help to guide future stratified treatment approaches. Given the difficulty of obtaining repeated skin biopsies in patients, minimally invasive sampling techniques such as tape stripping are a very promising approach to monitor disease biomarkers (129–133), but data in CTCL are still missing. Enlarged datasets will hopefully provide a better understanding of targetable disease subsets in this highly heterogeneous disease spectrum.

## Conclusion

While there is evidence of JAK/STAT activation in CTCL, its role in disease initiation and progression remains unclear. While mouse models are indispensable for better understanding basic mechanisms of inflammation and cancer, only clinical trials will be able to assess whether individual JAK or STAT alterations have a role in CTCL pathogenesis.
